# Assessing and managing frailty in emergency laparotomy: a WSES position paper

**DOI:** 10.1186/s13017-023-00506-7

**Published:** 2023-06-24

**Authors:** Brian W. C. A. Tian, Philip F. Stahel, Edoardo Picetti, Giampiero Campanelli, Salomone Di Saverio, Ernest Moore, Denis Bensard, Boris Sakakushev, Joseph Galante, Gustavo P. Fraga, Kaoru Koike, Isidoro Di Carlo, Giovanni D. Tebala, Ari Leppaniemi, Edward Tan, Dimitris Damaskos, Nicola De’Angelis, Andreas Hecker, Michele Pisano, YunfengCui, Ron V. Maier, Belinda De Simone, Francesco Amico, Marco Ceresoli, Manos Pikoulis, Dieter G. Weber, Walt Biffl, Solomon Gurmu Beka, Fikri M. Abu-Zidan, Massimo Valentino, Federico Coccolini, Yoram Kluger, Massimo Sartelli, Vanni Agnoletti, Mircea Chirica, Francesca Bravi, Ibrahima Sall, Fausto Catena

**Affiliations:** 1 Department of General Surgery, Singapore General Hospital, Singapore, Singapore; 2 Department of Orthopedic Surgery and Department of Neurosurgery, Denver Health Medical Center and University of Colorado School of Medicine, Denver, CO USA; 3 Department of Anesthesia and Intensive Care, Azienda Ospedaliero-Universitaria Parma, Parma, Italy; 4 John Hunter Hospital, University of Newcastle, Newcastle, NSW Australia; 5 Unit of General Surgery, San Benedetto del Tronto Hospital, av5 Asur Marche, San Benedetto del Tronto, Italy; 6 Department of Surgery, Denver Health Medical Center, Denver, CO USA; 7 Research Institute of Medical University Plovdiv/University Hospital St George Plovdiv, Plovdiv, Bulgaria; 8 Trauma Department, University of California, Davis, Sacramento, CA USA; 9 Faculdade de Ciências Médicas (FCM), Unicamp Campinas, Campinas, SP Brazil; 10 Department of Primary Care and Emergency Medicine, Kyoto University Graduate School of Medicine, Kyoto, Japan; 11 Department of Surgical Sciences and Advanced Technologies “GF Ingrassia”, University of Catania, Cannizzaro Hospital, Via Messina 829, 95126 Catania, Italy; 12 Oxford University Hospitals NHSFT John Radcliffe Hospital, Headley Way, HeadingtonOxford, OX3 9DU UK; 13 General Surgery Department, Helsinki University Hospital, Helsinki, Finland; 14 Department of Surgery, Radboud University Medical Center, Nijmegen, Netherlands; 15 General and Emergency Surgery, Royal Infirmary of Edinburgh, Edinburgh, UK; 16 Hôpital Henri Mondor, Université Paris Est, Service de Chirurgie Digestive et Hépato-Bilio-Pancréatique, Créteil, France; 17 Department of General and Thoracic Surgery, University Hospital, Giessen, Germany; 18 General and Emergency Surgery, ASST Papa Giovanni XXIII, Bergamo, Italy; 19 Department of Surgery, Tianjin Nankai Hospital, Nankai Clinical School of Medicine, Tianjin Medical University, Tianjin, China; 20 Department of Surgery, Harborview Medical Centre, University of Washington, Seattle, USA; 21 Department of Emergency Surgery, Centre Hospitalier Intercommunal de Villeneuve-Saint-Georges, Villeneuve-Saint-Georges, France; 22 General Surgery, Monza University Hospital, Monza, Italy; 23 3Rd Department of Surgery, Attikon General Hospital, National and Kapodistrian University of Athens (NKUA), Athens, Greece; 24 Department of General Surgery, Royal Perth Hospital, University of Western Australia, Perth, Australia; 25 Department of Trauma and Acute Care Surgery, Scripps Memorial Hospital La Jolla, La Jolla, San Diego, CA USA; 26 School of Medicine and Health Science, University of Otago, Wellington Campus, Wellington, New Zealand; 27 The Research Office, College of Medicine and Health Sciences, United Arab Emirates University, Al-Ain, UAE; 28 Department of Radiology, Tolmezzo Hospital, Tolmezzo, Italy; 29 General, Emergency and Trauma Surgery Department, Pisa University Hospital, Pisa, Italy; 30 Division of General Surgery, Rambam Health Care Campus, Haifa, Israel; 31 Department of Surgery, Macerata Hospital, Macerata, Italy; 32 Anesthesia and Intensive Care Unit, AUSL Romagna, M. Bufalini Hospital, Cesena, Italy; 33 Service de Chirurgie Digestive, Centre Hospitalier Universitaire Grenoble Alpes, Grenoble, France; 34 Healthcare Administration, Santa Maria Delle Croci Hospital, Ravenna, Italy; 35 Department of General Surgery, Military Teaching Hospital, Hôpital Principal Dakar, Dakar, Senegal; 36 Department of Emergency and Trauma Surgery, Bufalini Hospital, Cesena, Italy

**Keywords:** Emergency surgery, Laparotomy, Elderly, Frail, Frailty

## Abstract

Many countries are facing an aging population. As people live longer, surgeons face the prospect of operating on increasingly older patients. Traditional teaching is that with older age, these patients face an increased risk of mortality and morbidity, even to a level deemed too prohibitive for surgery. However, this is not always true. An active 90-year-old patient can be much fitter than an overweight, sedentary 65-year-old patient with comorbidities. Recent literature shows that frailty—an age-related cumulative decline in multiple physiological systems, is therefore a better predictor of mortality and morbidity than chronological age alone. Despite recognition of frailty as an important tool in identifying vulnerable surgical patients, many surgeons still shun objective tools. The aim of this position paper was to perform a review of the existing literature and to provide recommendations on emergency laparotomy and in frail patients. This position paper was reviewed by an international expert panel composed of 37 experts who were asked to critically revise the manuscript and position statements. The position paper was conducted according to the WSES methodology. We shall present the derived statements upon which a consensus was reached, specifying the quality of the supporting evidence and suggesting future research directions.

## Background

Many countries are facing an aging population. As people live longer, surgeons face the prospect of operating on increasingly older patients. As an example, in England, 2.5 million people above 75 years old, underwent surgery between 2014–2015, as opposed to just 1.5 million between 2006–2007 [1, 2]. Nearly 30% of these 2.5 million were over 85 years old [1]. Similarly, women aged 85 years and over now represent the largest proportion of emergency surgical admissions in Australia, compared with all other age and sex groups [3].

Traditional teaching is that with older age, these patients face an increased risk of mortality and morbidity, even to a level deemed too prohibitive for surgery. However, this is not always true. An active 90-year-old patient can be much fitter than an overweight, sedentary 65-year-old patient with comorbidities. Recent literature shows that frailty—an age-related cumulative decline in multiple physiological systems, is therefore a better predictor of mortality and morbidity than chronological age alone [4, 5].

Despite recognition of frailty as an important tool in identifying vulnerable surgical patients, many surgeons still shun objective tools. Many surgeons still choose to “eye-ball” their patients to determine fitness for surgery. However, this has been proven to be subjective and inconsistent between different observers [6].

Currently, there are more than 20 different frailty instruments in the literature [7]. However, in essence, these are derivatives of 2 main models of frailty. The first is the 'phenotype’ model described by Fried et al. [8]. Fried described frailty as a decline in lean body mass, strength, endurance, balance, walking performance and low activity. Patients who have three or more of the five features are deemed frail, while those who have none of the features are non-frail. Patients who display one or two of the five features are “pre-frail” [8].

The second model by Rockwood et al. is the Frailty Index (FI), or the cumulative deficit model, developed in the Canadian Study of Health and Aging (CSHA) [9]. This model conceptualizes aging as the accumulation of deficits and views frailty as a multidimensional risk state quantified by the number of deficits rather than by the nature of the health problems. An FI is based on a comprehensive geriatric assessment and is calculated as the number of deficits the patient has, divided by the number of deficits considered. The deficits encompass co-morbidities, physical and cognitive impairments, psychosocial risk factors and common geriatric syndromes [10]. The FI score ranges between 0 and 1, with higher scores indicating greater degree of frailty [11]. For example, in a frailty index based on a comprehensive geriatric assessment, an individual with impairments in 4 of 10 domains and with 10 of 24 possible co-morbidities would have 14 of 34 possible deficits, for a frailty index of 0.41.

Due to the developing literature over the last five years on the subject of frailty, there has been a change in who is thought of as ‘old’ [12]. Previous literature defined ‘geriatric’ as those above 65 years. However, that target is changing. In that regard, dedicated literature to studying frailty in the ‘old old’ and the ‘oldest old’ (aged 75–85 and over 85 years) is still lacking, especially in patients undergoing emergency laparotomy.

The aim of this position paper was to perform a review of the existing literature and to provide recommendations on emergency laparotomy and in frail patients. This position paper was reviewed by an international expert panel composed of 37 experts who were asked to critically revise the manuscript and position statements. The position paper was conducted according to the WSES methodology [13]. We shall present the derived statements upon which a consensus was reached, specifying the quality of the supporting evidence and suggesting future research directions.

## Methods and materials

### Search strategy

We searched seven electronic databases (Medline, CINAHL, The Cochrane Library, AMED, PSYCINFO, EMBASE and Web of Science) for manuscripts published from 1 January 1980 to 31 December 2022. All identified and relevant studies’ references were manually reviewed to identify any potential studies that met the inclusion criteria. The search terms were based on MeSH terms (Medical Subject Headings) and other controlled vocabulary. Search terms relating to surgery, frailty and risk factors were used.

### Eligibility criteria and study identification

Randomized controlled trials, cohort studies and case–control study designs were eligible for inclusion. Only studies using a validated method of frailty identification were included [14]. Studies based solely in intensive care were excluded since these populations are atypical and could introduce additional confounders. No language restrictions were applied.

### Data extraction and quality assessment

Demographics, frailty tools, frailty prevalence and outcomes data were extracted from the included studies. Prospective, randomized controlled trials and meta-analyses were given preference in developing these guidelines. The final grade of recommendation was performed using the Grades of Recommendation, Assessment, Development, and Evaluation system.

### Recommendations

#### 1a.

All patients being considered for, or undergoing emergency laparotomy aged 65 years or more, should undergo a routine frailty screening, e.g., Clinical Frailty Scale. Grade of recommendation: strong recommendation based on high-quality evidence, 1A.

#### 1b.

Vulnerable patients identified via the screening should receive a Comprehensive Geriatric Assessment (CGA) ideally upon admission, if not within 72 h of admission to a ward or step down from critical care to a ward. Grade of recommendation: strong recommendation based on high-quality evidence, 1A.

There are many frailty screening tools available. We recommend using a simple screening tool such as the Clinical Frailty Scale [9] which has been validated for use in patients. While CGA is the gold standard, this can be very time-consuming and inappropriate, especially if resources are limited or if the patient is very sick, hence a screening tool is a preferred choice upfront. [15].

A consensus group, consisting of 6-major international, European, and US societies, recommended administering routine frailty screening in all older people aged 70 years or older [16].

In the area of surgery, frailty is an independent risk factor for mortality, morbidity, length of stay, and postoperative complication [17, 18]. This has been well validated in the study of hip fractures, where frailty has been shown to be significantly associated with adverse outcome including mortality and length of hospital stay [19].

In the realm of trauma, a 15-item trauma-specific frailty index (TSFI) was validated from various centers. This TSFI, which consists of comorbidities, daily activities, health attitudes, sexual function, and nutrition domains, can also be assessed by the caregiver. It is an independent predictor of unfavorable discharge if greater than 0.27 [20].

The Guidelines from American College of Surgeons for Surgery and National Institute for Health and Care Excellence has emphasized frailty assessment in acute care settings or preoperative period as a new screening criterion for fitness [10, 19]. The 15-variable Emergency general surgery specific frailty index (EGSFI) was validated in a prospective cohort [21]. The EGSFI-based frailty status significantly predicted postoperative complications (odd ratio, 7.3; 95% confidence interval, 1.7–19.8), but age was not a relevant factor.

Similarly, the Emergency Laparotomy and Frailty [ELF] study showed an association between frailty and 90-day mortality (clinical frailty scale 5, OR 3.18, 95% CI 1.24–8.14), increased risk of complications and length of hospital stay in older emergency laparotomy patients [22].

One of the most important and scarce studies in the field of emergency laparotomy and frailty is the NELA audit [23]. The NELA audit [23] acknowledged that there are multiple frailty tools [24]. The audit adopted the Clinical Frailty Score and found it be prevalent in 11% of people over 65 years, rising to 50% in those over 85 years of age [23]. This score can also be applied to the younger surgical populations [25]. Therefore, the NELA proposed that the Clinical Frailty Score was the most appropriate screening tool for patients undergoing emergency laparotomy.

After screening the patients, the NELA audit utilized a cut off for Clinical Frailty Score of ≥ 5 (or aged ≥ 80 years with any frailty score), to identify patients who should receive geriatrician-led multidisciplinary comprehensive geriatric assessment (CGA) ideally upon admission, if not within 72 h of admission to a ward or step down from critical care to a ward [23].

The CGA has emerging evidence when applied to both elective and emergency older surgical populations [24–37]. Older emergency general surgical patients have been shown to benefit from perioperative CGA in terms of reduced mortality [28], LOS [27] and additional diagnoses and/or interventions made [29].

The CGA [30] is considered the benchmark for frailty assessment and generally includes follow-up care such as geriatric-specific optimization interventions [31]. The CGA addresses multidisciplinary components related to patients’ physical, mental, and psychosocial well-being and functional capabilities. However, it may be time-consuming to administer, and a geriatric assessment composed of questionnaires assessing different domains of well-being can often be used instead [32, 33]. Although most CGA studies for abdominal surgery have been performed for the elective setting and in oncology.

Feng et al. [36] performed a systematic review and showed that the CGA predicted surgical outcomes in 1019 patients who underwent a variety of elective oncologic operations. This study showed that dependency in instrumental activities of daily living (IADLs: preparing hot meals, grocery shopping, making telephone calls, taking medicines, and managing money), fatigue, and frailty were significantly associated with overall complications, and that dependency in IADL was predictive of discharge to an institutional setting (i.e., not the patient’s home). Although major complications were more frequent in patients with cognitive impairment and dependency in IADL and activities of daily living (ADL: walking, dressing, bathing, eating, getting into and out of bed, and toileting), age, per se, was not associated with a higher complication rate.

However, there are no studies on CGA when applied to emergency laparotomies in silo. Nevertheless, the depth of literature on CGA and outcomes in general shows that this can be considered. More research into this topic is needed. We also acknowledge that these studies used large databases and retrospective methodology that put more emphasis on the metrics of frailty obtained from a chart review (i.e., comorbidities and reported dependence) than on objective office-based frailty measures (i.e., grip strength and walking time).

Hence, the combination of frailty screening tools and the selective application of CGA is a good strategy. Using such screening tools to assess frailty preoperatively may help patients and their caregivers decide on a personalized treatment plan that aligns with their goals of care.

It is important to note that although studies on frailty and its impact on emergency laparotomy are limited, there are multiple studies on frailty and elective abdominal surgery. A multivariate analysis of a prospective study of 980 patients aged ≥ 75 years undergoing oncologic surgery demonstrated that frailty was associated with 6-month mortality after surgery (OR 1.14 for each unit increase in CGA score; *p* = 0.01). Interestingly, the ASA Physical Status Classification System Score, a commonly used marker of preoperative functional status, and age, was not associated with 6-month mortality in this study [37].

Similarly, a multivariate logistic regression analysis of 7337 patients from the American College of Surgeons National Surgical Quality Improvement Program (ACSNSQIP) who underwent elective colorectal cancer resection (mean age 65.8 ± 13.6 years) showed that frailty, assessed using an 11-point modified frailty index (m-FI), not age, was independently associated with readmission within a month of surgery (OR 1.4; 95% CI, 1.1–1.8) [38].

Meanwhile, another ACS-NSQIP study of 295,490 patients who underwent colorectal surgery for any indication between 2011 and 2016 showed that frailty, as assessed using a 5-item m-FI, was associated with significantly higher risks of prolonged length of stay (OR 1.24; 95% CI, 1.20–1.27), discharge to an institutional setting (OR 2.80; 95% CI, 2.70–2.90), 30-day serious morbidity (OR 1.39; 95% CI, 1.35–1.43), and mortality (OR 2.00; 95% CI, 1.87–2.14) [39].

#### 2.

Decisions regarding surgery should also consider patients’ degree of frailty rather than chronological age, as surgery may at times, result in poor outcomes. Grade of recommendation: strong recommendation based on high-quality evidence, 1A.

Neither surgeons nor patients have a true understanding about what their likely outcome is after emergency laparotomy surgery, particularly when frail. The recent Emergency Laparotomy in the Frail (or ELF) study had highlighted the deleterious effect of frailty on those who require an emergency laparotomy [22]. In nearly 1,000 consecutive patients aged over 65 years in 49 sites across England, Scotland and Wales, the ELF study showed that the presence of frailty was associated with greater risks of postoperative mortality. Their findings also demonstrated that as the degree of frailty increased, there was a linear increase in mortality.

The NELA audit [23] demonstrated that patients aged 65 years or over have worse clinical outcomes compared to their younger counterparts. These include longer length of hospital stay (median 15.2 days vs 11.3 days) and higher 30-day and 90-day mortality (15.3% vs. 4.9% and 20.4% vs. 7.2%). In addition, there is an association between frailty, which is known to increase with age, and increased 90-day mortality following emergency laparotomy (aOR for patients who were mildly frail and moderately/severely frail was 3.18 and 6.10), 1-year hospital visits (7.2 vs. 2.0) and care level (aOR for an increase in care level was 4·48 for vulnerable patients, 5·94 for those mildly frail and 7·88 for those moderately or severely frail, compared with patients who were fit). In NELA year 4, 6.7% of older patients compared with 1.9% (*P* < 0.001) of younger patients were discharged to care-home accommodation.

In general for patients who survived surgery, frailty is associated with worse surgical outcomes: increased mortality, prolonged hospital stay, complications and an increased level of social care provision on discharge from hospital [40]. Over 30% of older patients do not return to their own homes after emergency laparotomy surgery [41]. However, data highlighting long-term quality of life following emergency surgery are lacking, and epidemiological studies addressing this neglected area are desperately needed.

The research community are working hard to gather the evidence for the best interventions in this frail group of patients. The recent EPOCH study, a large-scale and well-designed step-wedged randomized controlled study of the implementation of a care pathway, in emergency abdominal surgery, showed no benefit of the intervention [42]. In the future, we may be able to personalize the intervention and better predict outcomes in this patient group.

#### 3.

Treatment plans for frail older adults should align with patients’ goals of care and should be based on a discussion regarding realistic outcomes. Grade of recommendation: strong recommendation based on low-quality evidence, 1C.

When contemplating the care plan for a frail patient, the goals of care should be discussed with the patient, engaged family, caregivers or advocates, and other members of the multidisciplinary team that may include representatives from surgery, geriatrics, palliative care, primary care, oncology, radiation oncology, and so on [43].

Typically, these discussions address domains such as anticipated longevity, functional status, independence, and comfort [44, 45]. In circumstances involving potential surgical intervention, deliberating whether to proceed with surgery should consider the likely treatment outcomes (including curative versus palliative objectives) and patient and family preferences.

A realistic picture should be presented based on the anticipated risks of morbidity, mortality, and cognitive decline for each of the proposed treatment options taking into consideration the patient’s unique presentation, degree of frailty, and functional status [46]. Specifically, patients may value their functional performance and cognitive status more than other treatment-related considerations and, as a result, patients may base their decisions on the likelihood of maintaining a certain level of performance.

Of note, the degree of cognitive decline associated with an individual surgery or anesthetic exposure is unknown. However, the Mayo Clinic performed a 5-year longitudinal study of 1819 patients aged ≥ 70 years and showed that exposure to general anesthesia and surgery was associated with subtle accelerated cognitive decline [47].

On an individual patient basis, it is important to clarify what matters most to patients‚ and online resources are available to facilitate these discussions (e.g., the American.

Geriatrics Society’s Health in Aging Foundation website, https://www.healthinaging.org/age-friendly-healthcareyou/ care-what-matters-most). In practice, it may be helpful to include a geriatrician and/or the patient’s primary care physician in treatment planning discussions. When planning operative treatment, it is helpful to clarify patients’ current living situation and existing support, to communicate goals for postoperative disposition as well as code status, and to have patients designate a surrogate decision-maker. Importantly, clinicians should recognize that patients’ goals of care may change during the perioperative period [42].

In the emergency setting, it may be difficult to have comprehensive goals of care discussions with patients, particularly if they are septic or unstable, have cognitive impairment, or are otherwise unable to have a meaningful discussion. An interdisciplinary, 23-member expert panel recommended a structured communication framework addressing 9 key elements to facilitate decision-making among seriously ill older patients with emergency surgical conditions [48].

The difficulties with having discussions in the setting of emergency circumstances highlight the importance of taking the opportunity to engage patients and their families in early goals of care discussions, especially when patients have multiple comorbidities or a condition that may result in a subsequent emergency (e.g., obstructing colorectal cancer) [49, 50].

#### 4.

Cognitive function should be assessed preoperatively routinely in frail older adults. Grade of recommendation: strong recommendation based on low-quality evidence, 1C.

The prevalence of dementia is estimated to be 5% among 70- to 79-year-olds, 24% among 80- to 89-year-olds, and nearly 40% among people older than 90 years [51]. Meanwhile, mild cognitive impairment (MCI) is distinguished from dementia in that the impairment is not severe enough to interfere with independent function. MCI is common among older adults, even those living independently, and affects up to 50% of patients older than 65 years [52].

Although the American College of Surgeons and the American Geriatrics Society recommend routinely assessing preoperative cognitive function and advocate using cognitive assessment tools such as the Mini-Cog preoperatively to detect MCI [53], the results of studies evaluating an association between MCI and postoperative outcomes such as complications, length of stay, and mortality are mixed and studies have been underpowered [54, 55].

Nonetheless, the most compelling reason to evaluate for cognitive impairment preoperatively is to predict and prepare patients and caregivers for the likelihood of postoperative delirium; preoperative MCI is one of the strongest predictors of postoperative delirium [56]. In patients found to have cognitive impairment, it is advisable, when feasible, to involve a geriatrician and/or psychiatrist and to implement delirium risk reduction interventions such as orientation to staff and surroundings, sleep hygiene, early mobilization, and optimization of vision and hearing [57–59].

In addition, decision-making capacity may be diminished in patients with cognitive impairment or dementia, and family members, health-care surrogates, and primary care physicians should be included in the decision-making process in appropriately selected patients [60]. Upon returning home postoperatively, patients with cognitive or memory impairment may benefit from close surveillance from caregivers or home care services.

Culley et al. studied 211 patients who underwent orthopedic surgery using the Mini-Cog‚ which includes a 3-item recall test and a clock-drawing task that tests visuospatial representation, memory, recall, and executive function. In this prospective study, 24% of the patients were identified with preoperative cognitive impairment (Mini-Cog score ≤ 2), which was associated with an increased postoperative incidence of delirium (21% versus 7%; OR 4.52; 95% CI, 1.30–15.68) [61]. Cognitive impairment, again measured using the Mini-Cog, was also observed in 21% of 1003 patients older than 70 years before undergoing major elective oncologic surgery in the prospective, multicenter Geriatric Oncology Surgical Assessment and Functional Recovery after Surgery study [62].

Another method for evaluating preoperative, baseline cognitive function is the 12-item Self-Administered Gerocognitive Examination‚ which detects MCI and early dementia among geriatric patients [63]. Benefits of the Self-Administered Gerocognitive Examination include its digital format‚ which can be administered while patients are in waiting rooms or even at home‚ and its ability to trend serial results over time [64].

#### 5.

Frail older adults should be actively screened for postoperative signs and symptoms of delirium and treated appropriately. Grade of recommendation: strong recommendation based on moderate-quality evidence, 1B.

Delirium, an acute confused state with hallmarks of fluctuating inattention and global cognitive dysfunction, occurs in up to 50% of older adults postoperatively [65] and may remain unrecognized in up to two-thirds of cases [66].

Delirium is associated with functional and cognitive decline, increased morbidity and mortality, longer lengths of stay, higher rates of nursing home placement, and increased health-care costs [67–73]. Moreover, as complications may present atypically in older adults, clinicians should recognize that postoperative delirium may be an indicator or manifestation of an underlying complication.

Maintaining an appropriate index of suspicion in frail older adults experiencing postoperative delirium and initiating a broad clinical workup under these circumstances may be advised (e.g., evaluating for infections, electrolyte abnormalities, and drug side effects) [74].

The Confusion Assessment Method screens for delirium by evaluating for (1) mental status changes with acute onset and fluctuating severity, (2) inattention, (3) disorganized thinking, and (4) an altered level of consciousness. Using the Confusion Assessment Method, the presence of 1, 2, and either 3 or 4 confirms the diagnosis of delirium [75].

Patients experiencing delirium may benefit from early geriatric or neuropsychiatric specialist consultation to assist with perioperative management as well as multimodal, nonpharmacologic interventions such as cognitive stimulation, early mobilization, preservation of the sleep–wake cycle, and ensuring adequate hydration [58, 59, 71].

Importantly, delirium can be prevented in up to 50% of patients by using a delirium prevention bundle [76]. Watt et al. performed a meta-analysis of 8557 patients older than 60 years who underwent elective orthopedic, cardiac, or abdominal surgery and found a pooled postoperative delirium incidence rate of 18.4% (95% CI, 14.3–23.3). In this study, the strongest predictors of postoperative delirium were a personal history of delirium (OR 6.4; 95% CI, 2.2–17.9), frailty (OR 4.1; 95% CI, 1.4–11.7), and cognitive impairment (OR 2.7; 95% CI, 1.9–3.8). In this study, prognostic factors that could potentially be modified to reduce the incidence of delirium included decreasing the use of psychotropic medications, smoking cessation, and increasing caregiver support [56].

Another intervention shown to decrease the incidence of delirium is avoiding or reducing the use of specific medications such as opioids, benzodiazepines, antihistamines, atropine, sedative hypnotics, and corticosteroids [77].

In 2019, in an effort to reduce adverse drug events in older patients and to decrease the incidence of delirium, the American Geriatric Society updated the Beers Criteria describing potentially inappropriate medication use in patients aged ≥ 65 years and specifically highlighted the detrimental effects related to antipsychotics, benzodiazepines, H2 receptor antagonists, anticholinergics, and meperidine [78].

#### 7.

Frail patients may benefit from a multidisciplinary approach to perioperative care that includes a health care provider with geriatric expertise. The provision of geriatric medicine support should be consultant-led throughout the patient pathway and integrated in the perioperative clinical care pathway. Grade of recommendation: strong recommendation based on low-quality evidence, 1C.

Geriatricians and practitioners with geriatrics expertise have specialized training and experience in assessing and managing geriatric syndromes (e.g., dementia, delirium, propensity for falling, comorbidity, and polypharmacy) and frailty and can improve the perioperative care of patients with these conditions. However, multidisciplinary approaches engaging these providers are commonly underused due to practice patterns and the limited number of available specialists [79].

Additionally, as previously mentioned, access to resources and support may limit the individual practitioner’s ability to engage a multidisciplinary team for the care of these patients. To supplement the work of geriatricians, practices can use other specialists, such as adult/geriatric nurse practitioners, social workers, nurse navigators, pharmacists, dieticians, rehabilitative medicine physicians, physical and occupational therapists, psychologists, and psychiatrists, to complete portions of the geriatric assessment and provide geriatric-related optimization [80].

Shahrokni et al. retrospectively studied the effects of geriatricians comanaging a cohort of 1020 patients who underwent cancer surgery for a variety of cancer types and required at least a 1-day hospital stay and compared this group to 872 similar patients who were treated with standard surgical service management (i.e., were not comanaged by a geriatrician). This cohort study found the adjusted probability of death within 90 days in the geriatric comanaged group was less than half the rate in the standard management group (4.3% vs. 8.9%; 95% CI, 2.3–6.9; *p* < 0.001). Although the 2 groups had similar complication rates, the geriatric comanaged group had greater usage of supportive care services (e.g., physical therapy, speech and swallow rehabilitation, and nutrition services), which may have contributed to the decreased mortality rate in this group. In addition, although not specifically studied, the geriatricians may have addressed risk factors for geriatric specific complications (e.g., risk for delirium and falls) [81].

In a similar study, 310 patients aged ≥ 70 years undergoing elective colorectal surgery were assigned to usual care (107 patients) or multidisciplinary, CGA-based care (203 patients) based on their preoperative comorbidities and level of independence. Although the patients in the multidisciplinary/CGA care group had more frequent serious complications (75.9% versus 56.1%; *p* < 0.001), as would be expected based on their comorbidities, patients in this group had a lower incidence of geriatric-specific complications (delirium 11.3% versus 29.2%; *p* < 0.001 and geriatric syndromes 10.3% versus 26.2%; *p* < 0.001) [82].

A pooled review of 12 studies that used variable methodology and included patients who underwent hip fracture surgery or emergency abdominal, trauma, and gastrointestinal surgery examined surgical outcomes among older adults and showed that hospital-based geriatric comanagement leads to shorter lengths of stay and lower mortality and readmission rates [83].

The NELA audit [23] showed that preoperative geriatrician review was recorded in 5.2% of older patients in year 4 of audit. Patients aged ≥ 85 years were more likely to receive geriatrician review; 20% postoperatively and 9.7% preoperatively. Preoperative geriatrician review in patients aged ≥ 65 years was associated with increased mortality (22.2 versus 13.3%, *p* < 0.001 30-day mortality; 27.9 versus 17.6%, *p* < 0.001 90-day mortality), whereas postoperative review was associated with reduced mortality (9.2 versus 16.1%, *p* < 0.001 30-day mortality; 17.2 versus 20.9% for 90-day mortality). All had a longer median time to theater (95.9 versus 32.2 h, *p* < 0.001 for preoperative review; 96.0 versus 37.3 h, *p* < 0.001 for postoperative review). This subgroup had no observed difference between baseline physiological and biochemical measures, however, were more likely to have predicted mortality risk ≥ 5% using all methods of risk assessment, be ASA grade 4 and have been admitted from a care home. Preoperative and postoperative geriatrician review was associated with increased LOS of 2.4 and 9.3 days, respectively, compared with older patients who did not receive geriatrician review. Covariates associated with discharge to care home included admission from care home (OR 11.113), age ≥ 85 years (OR 2.481) and postoperative geriatrician review (OR 2.329) [23]. Although geriatrician review is associated with reduced mortality in this cohort [23], only 24% of patients aged 70 years or over were assessed by a geriatrician following emergency laparotomy between December 2017 and November 2018 [84]. This low level of involvement of geriatricians reflects systemic issues, chiefly, the difficulty in recruiting geriatric medicine consultants. [85].

The United Kingdom has acted on the results of the NELA audit and is attempting to make systematic changes to improve this statistic. It is recognized that one of the drivers to improve the care of emergency laparotomy patients includes an enhanced best practice tariff (BPT) for delivery of specific care criteria known to be associated with improved outcome. In comparison, for hip fractures, UK national outcomes have improved following introduction of a BPT incentivizing geriatrician review, among other measures [86]. However, the current emergency laparotomy BPT criteria does not require geriatrician review.

Therefore, the British Geriatrics Society and Age Anaesthesia Association have established new standards of care for older people undergoing emergency laparotomy [87, 88]. Perhaps, the NHS England may implement these recommendations into the next iteration of the emergency laparotomy BPT as a driver to deliver evidence-based care for this high-risk group. This could be a good example for the rest of the world to learn and follow.

#### 8.

Frail older adults should be screened for social vulnerabilities and offered support. Grade of recommendation: strong recommendation based on low-quality evidence, 1C.

Social frailty, an incompletely explored concept, has been defined as a continuum of being at risk of losing or having lost resources that are needed to fulfill one or more basic social needs. Increasing age and lower levels of education are significant risk factors for developing social frailty [89, 90].

Considering that older adults rely on their social relationships and environment to effectively participate in multimodality care pathways, the concept of social frailty has become increasingly relevant to this vulnerable patient population [91].

Although this topic has been widely discussed, large prospective studies evaluating this concept especially in post laparotomies have not been reported. In an observational study, Hawkins et al. [92] evaluated 63 patients undergoing lower extremity amputation and showed that increased social integration (i.e., the number of contacts and interactions in a patient’s social network) was associated with improved postoperative function and QoL.

Another prospective study of 972 consecutive patients undergoing colorectal cancer resection showed that increased social support and decreased psychological distress improved health-related QoL 1 year after surgery [93]. A systematic review of 19 randomized trials by Gardner et al. [94] showed that providing practical social support was effective in enabling home-based health behavior change in frail older adults. In this study, patients with social support were more likely to have been instructed regarding positive behavioral changes and to have experienced appropriate changes in their environment; instituting positive behaviors such as using social services and following an individualized care path led to improvement in social functioning and general health.

#### 9.

Frail older adults should be managed with enhanced recovery protocols after surgery with modifications, as needed. Grade of recommendation: strong recommendation based on high-quality evidence, 1A.

Enhanced Recovery After Surgery (ERAS) is a multidisciplinary structured approach which provides standardized evidence-based components of care to patients undergoing specific types of surgery. To date, ERAS has largely been applied to elective surgery, but there is now evidence that high-risk surgical patients, such as those undergoing emergency laparotomy, can also benefit significantly from an ERAS approach [95–105]. These high-risk patients are likely to benefit from a structured approach with defined pathways of care and organizational resource allocation to prioritize their management [30, 106]. The ERAS for emergency laparotomy recognizes that this involves a diverse group of patients and therefore, needs specific programs for preoperative care, intra- and postoperative care, organizational aspects of management and end of life issues.

It must be recognized that the ERAS guidelines for emergency laparotomy are applicable for all patients with a non-elective, potentially life-threatening intra-abdominal condition requiring surgery. It, however, excludes trauma laparotomies, vascular conditions, appendectomy, and cholecystectomy.

Just under a decade ago, major cohort studies reported 30-day mortality for emergency laparotomy of between 14 and 18.5% rising to over 25% in patients over 80 years of age [107–109]. A review of patients with advanced cancer, who underwent emergency laparotomy for bowel perforation [110], showed a 30-day mortality of 34%; 52% of survivors were discharged to an institution. A number of studies have shown wide variation not only in outcomes, but also in the delivery of evidence-based care to this high-risk patient group [109–118].

Given the concerning nature of these outcomes, namely high patient morbidity and mortality, a number of groups worldwide started using evidence-based protocolized ERAS-like approaches in the management of these patients, with significant improvements in outcomes [95–99, 102, 118].

An equivalent to the ERAS, the NELA audit [23] has shown that outcomes have improved with 30-day mortality decreasing from 11.8 to 9.3% and performance on key process measures improving [119].

In spite of recent improvements, emergency laparotomy remains one of the highest risk surgical procedures with about one in ten patients deceased 30 days after surgery, rising to one in four over the age of 80 years [119]. Complications are common and mortality increases until at least 1 year [120]. Functional outcomes and return to independence can also be poor in survivors [121].

An ERAS approach has been shown to reduce mortality in patients over the age of 70 [95, 100]. A systematic review found an ERAS approach to be beneficial for older patients undergoing emergency surgery [101]. Many of these patients will also be frail, resulting in a lack of resilience in the face of a physiological insult [22, 122–124], and a validated frailty assessment [24, 125–127] should be performed if possible acknowledging the limitations in the acute environment.

Frail patients and those with cognitive impairment have a higher risk of mortality and morbidity which may not be captured by the commonly used surgical risk scores [24]. In a study of outcomes at 12 months in older patients after emergency laparotomy, the strongest predictors of mortality were frailty and increased American Society of Anesthesiologists (ASA) status [128].

Although ERAS is a dedicated system of enhanced recovery, there is much overlap with the perioperative CGA approach. Unlike ERAS in elective surgeries, where protocols are clear; ERAS is in emergency laparotomy that does not necessarily yet have clear pathways and protocols [129].

#### 10.

Minimally invasive techniques can be considered, where applicable. Depending on the patient’s condition and expertise of the surgical team. Grade of recommendation: strong recommendation based on low-quality evidence, 1C.

The benefits of minimally invasive surgery [MIS] include: decreased morbidity, shorter length of hospital stay, shorter time to resumption of a regular diet, decreased time to return of bowel function, decreased rates of postoperative pneumonia, reduced wound infection, reduction in patient’s surgical stress, reduced postoperative pain, early return to regular activities, cosmetic advantages and at times, better intraoperative visualization. These have been proven to be true especially in elective cases without any compromise to patient safety, oncological issues and operating room occupancy [130–139].

However, when MIS is applied to emergency surgery, the amount of available literature is poor in number in quality, resulting in lot of uncertainty. According to Surgical Societies guidelines and large retrospective studies with literature reviews, laparoscopic appendectomy, cholecystectomy and gastric and duodenal ulcer repair are well accepted emergency surgical procedures. However, their diffusion, even in the same hospital, can be influenced by insufficient expertise that may correlate with hospital organizational model. Other surgical procedures, such as laparoscopic treatment of small bowel occlusion, bowel resection for acute diverticulitis, are becoming more frequent, but they are still not routinely suggested [140–145]. In a recent report of a large observational study from UK, laparoscopy is adopted in less than 20% of major surgeries in emergency [146].

These difficulties of diffusion of minimally invasive surgery in emergency setting could be attributed to several reasons, i.e., more complexity when compared to elective surgery, sicker patients, higher level of diagnostic uncertainty, no regular day and week working hours, organizational issue, the lack of a dedicated surgical training and not homogeneous surgical and team skills.

According to the literature, laparoscopy is used in less than 20% of major emergency operations: the results of a recent research study from the National Emergency Laparotomy Audit (NELA) of England and Wales described that only 14.6% of cases were approached by laparoscopy with a conversion rate of 46.4% [146].

A research study from the USA reported an higher proportion of minimally invasive surgery in emergency general surgery (69.4%), but the majority of interventions were appendectomy and cholecystectomy: the proportion of major abdominal surgery in emergency performed with minimally invasive techniques was less than 20% [147].

Regarding major colorectal emergency surgery, several reports describe feasibility and safety; moreover, the promotion of the use of MIS is proved by lot of didactic articles [148–150]; however, in a large report, the proportion of patients treated with MIS was only 5.66% [148]. Data available in literature and the results of the present survey highlight an important need to improve the safe and effective use of minimally invasive techniques in emergency surgery.

The World Society of Emergency Surgery [129] performed a survey among surgeons. The survey identified that the main positive factors that could led to a higher uptake of MIS in emergency surgeries were the longer personal experience and the use of laparoscopy in elective surgery. Similarly, expertise in bariatric surgery and prevalent use of laparoscopy in major abdominal surgery were directly related to the use of laparoscopy in emergency surgery [129].

Dedicated training in emergency laparoscopic surgery and initiatives of continuing professional development may be beneficial in order to be able to offer the advantages of mini-invasive approaches to a larger number of patients. A surgeon with more developed skills in elective surgery and more experienced in elective laparoscopic surgery is more prone to use laparoscopic surgery also in primary emergencies. On the contrary, trauma surgery usually requires dedicated teams with specific skills [151, 152] that may not include minimally invasive techniques.

Among patients’ conditions, the only factors that seem to be limiting factors in the use of MIS in emergency surgery is the shock condition, and high predicted morbidity and mortality according to the most common clinical scores such as ASA, P-POSSUM and APACHE II [129]. As MIS in emergency surgery is in itself a developing field, the exact study of MIS in particularly frail patients is lacking.

#### 11.

In older adults, patient-centered postoperative functional outcomes and discharge destination planning should be considered in addition to traditional postsurgical outcomes. Grade of recommendation: strong recommendation based on moderate-quality evidence, 1B.

Outcomes after surgery have been measured by several indicators such as length of stay, morbidity, mortality, overall survival, disease-free survival, as well as time to first flatus or time to first oral intake. However, many of these metrics may have limited relevance to frail older adults who may be more concerned about anticipated disability and dependence than even a cancer diagnosis or limitation in life expectancy.

Banks et al. [153] analyzed data from a survey of 89,574 Australians (age > 45 years) and found that, although 7.5% of all respondents suffered from high levels of psychological distress, those with cancer and disability attributed stress much more strongly to their level of disability than to their cancer diagnosis. Another study published by a social research institute was based on surveys, found that although longevity may be the most important priority for most patients, this notion changes for older, retired patients who rank continued independence as important as maintaining health [154].

Functional recovery may incorporate organ-specific postoperative outcomes and patients’ ability to regain preoperative functional status. Loss of independence, or an increase in support required by patients after hospital discharge, is an example of a relevant functional recovery metric used to evaluate frail older patients.

Regaining independence, typically measured as a composite outcome, includes an assessment of cognition and nutritional status and the ability to perform routine ADLs and to walk proficiently [62]. Although a variety of instruments have been proposed to evaluate these kinds of domains, the most compelling literature uses the ADL, Mini-Cog, and Timed Up and Go/6-min walk distance scores [24, 72, 155–159].

De Roo et al., in a retrospective matched cohort study, highlighted the importance of analyzing less conventional outcomes like functional decline‚ which was defined as an increase in the number of ADLs requiring assistance after surgery. In this study, 289 patients who underwent colorectal surgery and were older than 65 years were compared to 867 control patients who did not undergo surgery [155]. De Roo et al. found that patients who underwent surgery and had a complication (90 patients, 31% of the surgical cohort) had a higher likelihood of functional decline (OR 2.96; 95% CI, 1.70–5.14) compared to controls and those who did not have a surgical complication (OR 1.82; 95% CI, 1.22–2.71) [155].

Patient-reported outcomes (PROs), despite their complexity, can be captured by integrating smart devices into electronic medical record systems and using a variety of programs to collect data [160, 161]. The University of California, Los Angeles 3-Item Loneliness Scale, evaluating how often patients feel they lack companionship or feel left out or isolated from others, was also reported to be useful in assessing PROs in frail individuals [162].

#### 12.

Current adoption of holistic CGA-based care for patients is dismal and systems need to improve and catch up and actions must be taken for implementation. Grade of recommendation: strong recommendation based on moderate-quality evidence, 1B.

While the benefits of coordinated multidisciplinary care of older surgical patient have been demonstrated and geriatric surgical liaison as routine care for all older laparotomy patients is currently recommended by NELA as best practice, the delivery of this care is still very variable across the world. Even in a dedicated NELA audit [23], 23% of patients over 70 years received geriatric involvement following their laparotomy [22]. On a more positive note, a recent survey of provision of surgical care for older people showed that the picture has improved over recent years, in all areas of surgical liaison, including general surgery, and hopefully this trend will continue [163].

Multiple factors are likely to have contributed to these improvements; a greater awareness of outcomes, hospital-level benchmarking data, publication of standards by professional stakeholders, quality improvement initiatives and focused education and training [161, 164, 165].

With the United Kingdom as a good example, there has been a shift toward identifying high-risk patients with a predicted 30-day mortality risk ≥ 5% and providing targeted interventions for this cohort, informed through the development of the tailored NELA risk model [23, 163] and the High-Risk General Surgical Patient Guideline (HRGSP) [178,179. Acknowledging that the majority of patients in the high-risk category are older, this guideline advocates proactive identification of frailty and supports novel collaborative partnerships between general surgery and geriatric medicine alongside traditional clinical stakeholders. Despite these initiatives, consistent challenges in implementation remain, in terms of pathway development, workforce and funding [163, 164].

#### 13.

Special consideration for patients above 90 years old, where morbidity and mortality rates may be high and natural life span is limited. In selective cases, conservative management is acceptable. Grade of recommendation: strong recommendation based on low-quality evidence, 1C.

With the steady rise in life expectancy and the associated growth in the number of people living to 90 years and beyond, patients at the extremes of old age are increasingly presenting with surgical pathology. As these individuals often have significant co-morbidities and disability limiting quality of life, it can be difficult to determine which patients would see significant benefits from undergoing an operation given its potential substantial risk. Counselling patients and their families regarding risks and benefits is complex because it is difficult to prognosticate in this age group owing to the lack of data.

When considering overall operative mortality (combining elective and emergency cases), Sudlow et al. [165] reported a 30-day mortality rate of 6.2%. Hosking et al. reported similar overall operative mortality at 8.4% [185]. The 30-day mortality rate for emergency operations studied by Pelavski et al. was 35.3% [186] compared with 17.4% for emergency patients in the study by Hosking et al. [185] and 9.7% by Sudlow et al. [165].

Not surprisingly, the most striking differences were noted when comparing elective and emergency surgery. Sudlow et al. [165] reported that the 30-day mortality rate for elective procedures in all operative categories was 0% versus 9.7% in the emergency cohort (*p* = 0.014). The difference in outcome was even greater when considering mortality at 90 days (5.2% elective vs 19.4% emergency, *p* = 0.013) and persisted even beyond that time.

Having an emergency procedure was also associated with more patients requiring higher level care in the postoperative period. Sudlow et al. [165] showed complications and morbidity were higher for the emergency group (57.0% vs. 13.7%), and these tended to be more serious. These findings are similar to rates observed for emergency cases by Hosking et al. [185] and Racz et al. [187].

Sudlow et al. [165] showed that almost half (46.2%) of the patients who underwent a major procedure died within 30 days. The mortality rate rose to 53.8% at 90 days and 61.5% at 1 year. There was a significant difference in overall median survival compared with patients who required less severe operations. Those undergoing major surgery had mortality rates increase from 7.0 at 30 days to 11.6% at 90 days and 20.9% at 1 year while other procedures had mortality rates increase from 2.1 to 17.0 to 25.5%, respectively.

Such high mortality rates are a serious issue. The rise in mortality over time could suggest that the perceived benefit of surgery might not be as prolonged as initially hoped. This calls into question the value of surgical intervention for these cases.

One of the most difficult aspects of deciding whether an individual should undergo any type of surgical intervention is determining whether he or she will benefit from the procedure, or whether it may actually cause harm or prolong suffering unnecessarily when there is no remaining quality of life.

The majority of surgeons believe that prolonging the life of a patient with poor functional/mental capacity serves no purpose, and this issue is commonly at the forefront of a decision of surgeons, patients and families not to proceed with surgery.

Even in individuals with relatively few co-morbidities, these patients still have a relatively limited life expectancy. For example with a 90-year-old woman in England expected to live a further 4.50 years and a man 3.94 years [188]. The decision to operate on anyone aged over 90 years should take into account not only the outcomes presented in this paper but also the very limited life expectancy of these patients. In fact, the surgeon should also consider patient factors, the nature of the procedure itself and the operative setting as well as the life expectancy. The decision in emergency cases is often one of life or death. In these circumstances, patients, their families and the operating surgeon may be willing to accept a higher level of risk, irrespective of whether this is necessarily the right thing to do.

## Conclusions

Major abdominal surgery in an emergency setting carries with it a high morbidity. Patient selection and identification of those who will likely benefit from surgery is difficult in the frail and elderly owing to the combination of multiple co-morbidities and the general decreased physiological reserve associated with aging. As highlighted in the National Confidential Enquiry into Patient Outcome and Death report on surgery in the elderly, identification of frailty is a key factor in case selection [13]. Frailty should be considered an independent risk factor for poor surgical outcomes. However, assessment of frailty is in itself difficult. Clear and frank information about the significant risks associated with surgery in elderly patients must be discussed with patients and their families to help set realistic expectations.

## Data Availability

Not applicable.
